# 863 Use of a Dual-Layer Biosynthetic Skin Substitute as a Temporizing Measure Prior to Autografting

**DOI:** 10.1093/jbcr/iraf019.394

**Published:** 2025-04-01

**Authors:** Lyndsay Deeter, Karen Richey, Kevin Foster

**Affiliations:** Diane & Bruce Halle Arizona Burn Center; Diane & Bruce Halle Arizona Burn Center; Diane & Bruce Halle Arizona Burn Center

## Abstract

**Introduction:**

Early debridement of full thickness burns is the standard of care. Frequently, these injuries are not amenable to grafting at their initial debridement and require temporizing measures. Traditionally, cadaveric allograft has been the mainstay of temporary wound closure until more definitive closure can be obtained. With time, temporizing techniques have evolved. Biosynthetic options are now available and published studies have alluded to them being comparable to cadaveric allograft in this patient population. Utilization of one such biosynthetic option was started in our burn unit recently, and our goal is to provide a case series to evaluate its safety and efficacy.

**Methods:**

This is a retrospective case series of patients suffering a full-thickness thermal injury who were treated with a two-layer biosynthetic skin substitute (BSS). The review spanned a 3-month period. Following tangential excision and debridement, all received BSS as a temporizing measure. Patients who died were excluded. Basic demographic, injury, surgical and hospital data were collected. Descriptive statistics were calculated.

**Results:**

A total of 10 patients are included in this analysis. The mean age was 46 years (range 1.42-74 years) and the majority (60%) were male. Average TBSA was 8.9% (range 1.5-25.5%) and 70% suffered a flame burn and the remaining 30% suffered a scald injury. Two patients required critical care with an average of 57.5 ventilator days, 61.5 ICU days, total length of stay for the entire cohort was a mean of 28.5 days (range 3-103). No patients received allograft or a other temporizing measure to their wound prior to the application of BSS. Eight of the ten subjects had isolated wounds that were treated solely with BSS, the average time from BSS placement to split-thickness skin grafting (STSG) was 3.6 days. Two of these 8 experienced graft failure that required repeat STSG.

**Conclusions:**

This case series demonstrates that BSS is a safe and viable alternative to cadaveric allograft. Both provide adequate, temporary coverage to a thermal injury in full thickness burn injuries following excision prior to placement of a split thickness skin graft. However, it appears as though BSS allows for a shorter time from excision to autografting and wound closure. The implications for cost, length of stay and pain control for patients who achieve wound closure in a shorter length of time warrants further investigation.

**Applicability of Research to Practice:**

A prospective randomized trial comparing cadaveric allograft to BSS is warranted and may result in a change to treatment paradigms.

**Funding for the Study:**

N/A

